# Salivary Amylase Gene Copy Number Relates with BMI Z-Score and with Response to Lifestyle Intervention for Children with Overweight and Obesity

**DOI:** 10.3390/ijms26189059

**Published:** 2025-09-17

**Authors:** Margalida Monserrat-Mesquida, Cristina Bouzas, Hélia Cardoso, Silvia García, Emma Argelich, David Mateos, Monica Marques, Catarina Campos, Elsa Lamy, Josep A. Tur

**Affiliations:** 1 Physiopathology of Obesity and Nutrition (CIBEROBN), Instituto de Salud Carlos III, 28029 Madrid, Spain; 2 Health Research Institute of Balearic Islands (IdISBa), 07120 Palma, Spain; 3 Research Group on Community Nutrition and Oxidative Stress, University of the Balearic Islands-IUNICS, 07122 Palma, Spain; 4 MED—Mediterranean Institute for Agriculture Environment and Development, University of Évora, 7004-516 Évora, Portugal

**Keywords:** childhood obesity, BMI z-score, *AMY1* gene, saliva, dietary intake, lifestyle intervention

## Abstract

The prevalence of childhood obesity has risen significantly, with numerous associated health risks. Emerging research suggests a potential role for genetic factors; particularly, copy number variations (CNVs) of the *amylase 1* gene (*AMY1*) may influence obesity through dietary behavior and metabolic regulation. This study aimed to examine the relationship between BMI z-score, dietary intake, and salivary *AMY1* gene copy number (CN) in children with overweight and obesity, and to assess the relationship between *AMY1* CN and the impact of lifestyle intervention on these parameters. The study included 90 children aged 2–6 years with overweight or obesity. Participants were randomized into either a parent support intervention group or a standard care control group. Anthropometric measurements, dietary intake, and salivary *AMY1* gene copy number were assessed at baseline and after a 9-month intervention. Positive correlations were found between *AMY1* gene copy number, BMI z-score, and carbohydrate intake, suggesting a potential role of this gene in dietary behavior-related obesity. The parent support intervention led to significant reductions in children’s BMI, BMI z-score, and energy and macronutrient intake compared to standard care. Although there was no direct association between *AMY1* copy number and changes in BMI z-score, higher *AMY1* copy numbers were associated with greater reductions in polyunsaturated fatty acid intake. These findings highlight an interaction between salivary *AMY1* gene copy number, dietary intake, and obesity in children. These results support the relevance of genetic factors in obesity-related dietary patterns and emphasize the effectiveness of targeted family-based lifestyle interventions.

## 1. Introduction

The prevalence of overweight and obesity in children and adolescents has increased exponentially in recent years. According to the latest data from the World Health Organization (WHO), around 37 million children under the age of five are classified as children with overweight in the world. Furthermore, over 390 million children and adolescents aged 5 to 19 are overweight, with 160 million of them classified as children with obesity [1]. For children under five years of age, overweight is defined as a weight-for-height measurement exceeding two standard deviations, based on the WHO Child Growth Standards [2], whereas obesity is defined as having a weight-for-height measurement that exceeds three standard deviations above the median according to the WHO Child Growth Standards [3]. Spain ranks among the European countries with the highest rates of childhood overweight and obesity, affecting approximately 39% and 18% of children aged 6 to 9, respectively [4].

Certain stages in childhood are considered critical periods for obesity prevention due to significant changes in body composition. These include the first two years of life, the adiposity rebound period (between ages five and seven), and puberty [5]. Early childhood is particularly important, as increases in body mass index (BMI) typically begin around age four or five. Evidence suggests that children with overweight at five years old are four times more likely to become obese later in life [6]. Once established, excess weight is difficult to reverse and substantially increases the risk of adult obesity, highlighting the importance of early intervention [7].

Childhood obesity is associated with numerous complications, including lipid metabolism disorders, hypertension, hyperinsulinemia, hepatic steatosis, obstructive sleep apnea syndrome, chronic inflammation, and psychological issues [8]. To monitor weight changes in children, BMI z-scores are commonly used, as they account for age and sex differences [9]. In short-term intervention studies, changes in BMI z-scores, as defined by the Centers for Disease Control and Prevention (CDC) growth charts, are frequently used as the primary outcome measure [10].

Carbohydrates are the main dietary factor influencing insulin secretion and postprandial glycemia, and they have been linked to the development of metabolic diseases [11]. Although many factors affect glucose homeostasis, recent research suggests that not only pancreatic but also salivary amylase may play a role in glucose regulation [12]. Salivary α-amylase, encoded by the *amylase 1* gene (*AMY1*), is secreted by the salivary glands and initiates carbohydrate digestion in the oral cavity. It breaks α-1,4-glycosidic bonds in starch to produce maltose, maltotriose, and eventually glucose [13]. Additionally, salivary amylase is thought to influence the oral perception of starchy foods [14] and sweetness [15], further supporting its role in food intake regulation.

The *AMY1* gene shows low variability in single nucleotide polymorphisms (SNPs) but high interindividual variability in copy number. Since gene copy number is directly associated with protein expression, salivary amylase levels vary widely among individuals [16]. The literature presents conflicting findings on the relationship between *AMY1* copy number variation (CNV) and obesity [16,17,18]. Some studies made in adults report a negative association between CNV and obesity [16] while others found no such relationship [19,20,21]. In children, a negative association between *AMY1* CNV and obesity was reported in some studies [17], although other researchers observed no association between the number of copies of this gene and childhood obesity [22]. However, studies have been performed in different populations, with different genetic backgrounds, and in this last study, when performing a metanalysis, to increase the sample number, a significant negative association between AMY1A CN and obesity, in Mexican children, was observed. Moreover, several studies suggest a link between the *AMY1* CNV and habitual starch intake [23,24,25]. As such, these inconsistencies may be partly due to differences in population age, ancestry, and dietary intake.

Given the lack of consensus regarding the relationship between *AMY1* CNV, diet, and obesity, the current study aimed to assess the association between salivary *AMY1* gene copy number, BMI z-score, and dietary intake in children with overweight and obesity. Additionally, the study assessed the effects of lifestyle intervention on these parameters, as well as how *AMY1* CNV impacted intervention responses.

## 2. Results

Table 1 shows the anthropometric characteristics and macronutrient intake of participants at baseline, stratified by BMI z-score tertiles. Significant differences were observed in weight, waist circumference, and BMI across tertiles, with children in Tertile 3 showing higher values compared to those in Tertile 1. No differences were found in the intake of major macronutrients. On average, carbohydrates, proteins, and total fats contributed approximately 46%, 16%, and 34% of total daily energy intake, respectively, across all participants.

**Figure 1:**
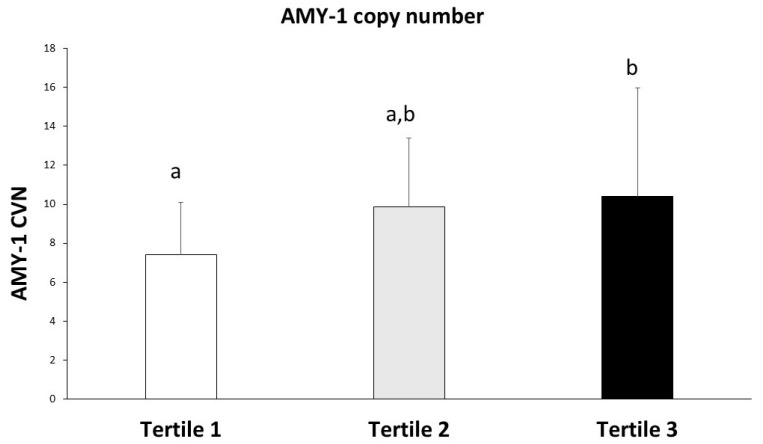
*AMY1* copy number according to z-score BMI. Statistics realized by one-way ANOVA and post hoc DMS. Different letters mean significant difference when *p* < 0.05.

Figure 1 displays the distribution of AMY1 gene copy number across the BMI z-score tertiles. The mean AMY1 copy number was 7.42 (SD = 2.67) in Tertile 1, 9.86 (SD = 3.52) in Tertile 2, and 10.4 (SD = 5.56) in Tertile 3. The mean *AMY1* copy number was higher in Tertile 3 compared to Tertile 1. Although differences between Tertiles 1 and 2 and between Tertiles 2 and 3 were not significant, the difference between Tertiles 1 and 3 reached significance. A weak but significant positive correlation was found between *AMY1* copy number and BMI z-score (r = 0.253, *p* < 0.05), suggesting that children with higher BMI z-scores tended to have a greater number of *AMY1* copies (Table 2).

It is also observed that there was a tendency for a correlation between *AMY1* copy number and the intake of fat, with a statistically significant and moderate correlation with PUFA intake (before intervention).

Although age or gender did not have effect on this correlation, controlling for total energy intake increased the correlation to r = 0.322 (*p* = 0.020).

Table 3 summarizes the changes in anthropometric measurements and dietary intake after the 9-month lifestyle intervention, comparing the standard care group with the parent support program group. Significant reductions in BMI and BMI z-score were observed in children after nine months in the parent support program group. Furthermore, significant decreases in energy, carbohydrates, total fat, and SFA intake were found only in the intervention group after nine months.

To assess the potential relationship between *AMY1* copy number and the intervention’s effects, and as no significant associations were found in the control group, Table 4 shows the correlation between *AMY1* copy number and changes in anthropometric and macronutrient intake after nine months of treatment in the parent support program group. A significant negative correlation was found only for PUFA intake (r = −0.448; *p* < 0.05), showing that children with higher *AMY1* copy numbers experienced greater reductions in PUFA intake. No significant associations were observed for changes in carbohydrate, total fat, or SFA intake.

## 3. Discussion

The main findings of this study show a significant relationship between salivary *AMY1* gene copy number, dietary intake, and obesity in children. Differences in anthropometric characteristics and dietary intake were observed across groups stratified by BMI z-score. Specifically, children in the highest tertile (Tertile 3) showed significantly higher body weight and waist circumference compared to those in the lowest tertile (Tertile 1). These results align with the expectations, as BMI z-score is a standardized indicator of adiposity relative to age and sex, and higher values are associated with increased body fat and related anthropometric measures [26,27].

Interestingly, despite these anthropometric differences, no significant variation in total energy or macronutrient intake was observed across BMI z-score tertiles. These findings suggest that factors beyond total nutrient intake, such as food quality, metabolic efficiency, energy expenditure, or gut microbiota, may contribute to differences in BMI among young children [28,29,30,31].

Salivary α-amylase, the enzyme responsible for initiating starch digestion, has been linked to both metabolic processes and dietary preferences [32]. In the current study, children with higher BMI z-scores had a higher number of *AMY1* gene copies, showing a potential association between salivary *AMY1* expression and increased adiposity. The previous literature on this topic is inconclusive; while some studies report a negative association between *AMY1* copy number and obesity [16,18], others find no significant relationships [19,20]. It appears that the relationship between *AMY1* gene copy number and obesity may depend on other factors, such as sex [33], reporting a negative relationship between *AMY1* CN and children obesity only in male, and ethnicity [32], showing higher strength in negative association between *AMY1* copy number and obesity in American African that in American European. Moreover, the usual starch intake also seems to be considered when relating the copy number of this gene with BMI. It was previously pointed out that in people with low dietary starch intake, a higher *AMY1* copy number appears to be protective of obesity, whereas in people with high starch intake, it is the opposite [34].

The current findings further contribute to this discussion by demonstrating a positive correlation between *AMY1* copy number and dietary intake. This suggests that children with more copies of the *AMY1* gene may be more inclined toward energy-rich diets. Although there were no statistically significant correlations with carbohydrate intake (what might be due to the lack of distinction between simple and complex carbohydrates in the present evaluation), it is not possible to exclude that the tendency for positive correlation with the total energy consumed can also reflect also higher intake of starch [35]. Moreover, *AMY1* copy number was also positively associated with PUFA intake. This correlation may reflect broader dietary behaviors or food preferences, warranting further investigation into the gene’s potential influence on fat metabolism [36].

The intervention results support the effectiveness of the parent support program in promoting weight reduction and improving dietary habits. Children in the intervention group experienced significant decreases in BMI, BMI z-score, and intake of energy, carbohydrates, total fat, and saturated fat, compared to those in the standard care group. These findings are consistent with previous research highlighting the value of family-based and behavior-focused interventions in managing childhood obesity [37,38,39]. Additionally, the parent support group showed significant reductions in energy, carbohydrates, fat, and saturated fat (SFA) intake, suggesting that dietary changes were an important part of the intervention [40,41,42]. These reductions in energy and macronutrient intake are likely contributing factors to the observed decreases in BMI and z-score BMI [43,44,45].

The change in PUFA intake during the intervention was negatively correlated with *AMY1* copy number. That is, children with more *AMY1* copies exhibited greater reductions in PUFA intake over the 9-month period. Since the correlation between *AMY1* copy number and PUFA, measured at the beginning of the experiment, was positive (i.e., higher PUFA intake in children with higher number of copies), this may suggest that, to achieve nutritional recommendations, these children had higher reductions in fat intake. These findings may reflect a shift in dietary preferences or adherence to nutritional recommendations prompted by the intervention. Alternatively, it could suggest a more complex metabolic interaction between *AMY1* expression and lipid processing, which should be explored in future studies [46].

Although AMY1 copy number was associated with BMI z-score in our cohort, this finding contrasts with some reports showing either stronger or null associations. We did not detect a modifying effect of sex, as the AMY1×sex interaction was not significant, suggesting similar associations in boys and girls. Our sample was ethnically homogeneous (European origin), which limits the possibility of exploring ethnic differences, a factor that has been suggested to influence the relationship between AMY1 copy number and obesity risk in more diverse populations. The different methodological approaches, including the molecular techniques used (e.g., qPCR vs. digital PCR) must also be not excluded as potential sources of slight differences among studies.

### Strengths and Limitations

As a strength of this paper, it could be highlighted that the study design included both anthropometric measurements and dietary intake data, allowing for an in-depth analysis of the relationship between *AMY1* copy number, diet, and BMI z-scores. By implementing a 9-month intervention, the study was able to assess changes over time (longitudinal), offering insights into the effectiveness of dietary and lifestyle interventions. The inclusion of *AMY1* gene copy number analysis adds a novel dimension to the study, linking genetic predispositions to dietary patterns and obesity outcomes in children. The recruitment of overweight/obese participants from different z-score BMI tertiles ensures that the findings are representative across a spectrum of overweight and obesity levels. At the same time, by not including normal weight children, we were able to study a relatively homogeneous sample, reducing variability unrelated to excess adiposity. The study adhered to ethical guidelines and used validated tools for data collection, such as standardized questionnaires and reliable PCR methods for genetic analysis.

This paper has some limitations too. The relatively small sample size (*n* = 90, reduced by the length of duration of the intervention) may limit the generalizability of the findings to broader populations. Moreover, the generalization of the findings to the full pediatric population cannot be made, since we studied only overweight and obese children, and we recognize that *AMY1* CN–BMI relationships may look different across the entire BMI spectrum. While the intervention lasted nine months, longer follow-up periods are necessary to evaluate the sustainability of BMI reductions and dietary changes. Although the study accounted for many variables, factors such as physical activity levels and socioeconomic status, which can influence BMI, were not fully controlled or analyzed. The focus on children aged 2–6 years may restrict the applicability of the findings to other age groups, such as adolescents, but at the same time it allows higher homogeneity of the studied group, reducing the influence of factors such as puberty.

## 4. Materials and Methods

### 4.1. Study and Participants

A total of 90 children over 2 to 6 years, classified as having overweight or obesity according to international cut-off criteria [47] and residing in the Balearic Islands (Spain), were included in the STOP study (The Science and Technology in Childhood Obesity Policy project). Inclusion criteria required that children had no underlying medical conditions, had not started any treatment for overweight or obesity, and that at least one parent was able to communicate with the recruitment center.

The study protocol adhered to the Declaration of Helsinki and was approved by the Balearic Islands Ethics Committee (ref. IB 3814/18 PI). Further protocol details are available on ClinicalTrials.gov (ref. CTN03800823) [48]. After providing written informed consent, participants were randomly assigned (1:1) to either the intervention group (parent support program and mHealth booster) or the control group (standard care based on national protocols). Participants allocated to the intervention group received the More and Less Europe (ML Europe) program, a structured parent-support intervention aimed at promoting healthy lifestyle behaviors in preschool children. The program was delivered through group sessions with parents, focusing on nutrition, physical activity, sleep routines, and behavioral strategies to support sustainable lifestyle changes in the family environment. To reinforce adherence at home, the program was complemented with a mobile health (mHealth) booster, consisting of a mobile application that provided tailored reminders, motivational messages, and tips related to healthy habits. Participants in the control group received standard care based on national pediatric protocols. A full description of the intervention is available in Ek et al. [49].

**Figure 2:**
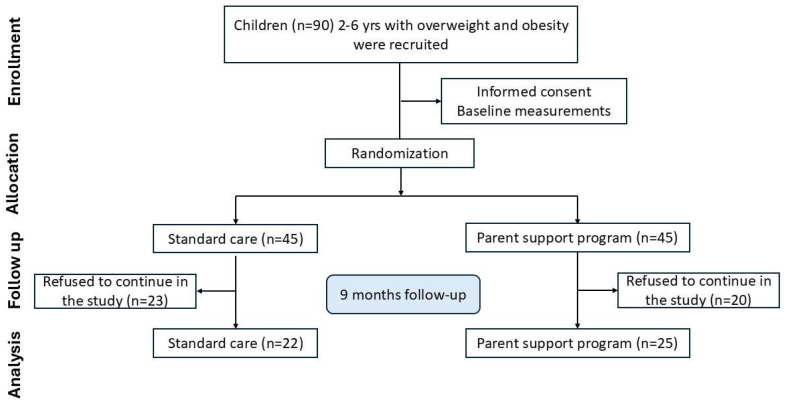
Flow-chart of study.

Following the 9-month intervention [49], 47 children who attended the follow-up visit were retained for analysis: 22 in the standard care group and 25 in the parent support group (Figure 2). Participants discontinued their involvement in either the intervention or control group mainly due to lack of time, changes in personal circumstances, health-related issues, or loss of interest.

### 4.2. Anthropometric Parameters

Children’s weight and height were measured to the nearest 0.1 kg and 0.1 cm, respectively. Height (m)was assessed using a fixed stadiometer (Seca 213, SECA Deutschland, Hamburg, Germany), and weight (kg) was measured with the child wearing only underwear using a segmental body composition analyzer in accordance with the manufacturer’s instructions (Tanita BC-418, Tanita, Tokyo, Japan). BMI was calculated as weight in kilograms divided by the square of height in meters (kg/m^2^). Age- and gender-specific reference values were then used to compute BMI z-scores [47].

### 4.3. Dietary Assessment

Participants were instructed to complete three 24 h dietary recalls on non-consecutive days, including one weekend day, during in-person interviews conducted by trained dietitians. The 24 h recall method is considered a gold standard in dietary assessment [50]. The recalls were carried out on-site with support from a dietitian, who verified the records immediately to confirm their precision and completeness. Two of the recalls referred to weekdays (Monday–Thursday), while the third captured eating behavior on a weekend day (Friday, Saturday, or Sunday). Each recall gathered detailed information regarding meal timing, eating setting, types of foods consumed, and portion sizes. Portions could be reported using familiar household units (e.g., cups, teaspoons, tablespoons) or specified in grams (g) and milliliters (mL). To estimate nutrient intake, the reported frequency of consumption was multiplied by the nutrient content of each portion. Average daily intakes were then obtained by distributing these values across the seven days of a typical week [51]. Dietary data were analyzed using EvalFINUT^®^ version 2.0 (FINUT, Granada, Spain), which is based on the Spanish Food Composition Database (BEDCA) and the USDA food database. This software converted reported food intake into daily estimates of energy and nutrient consumption, expressed in kilocalories (kcal), grams (g), milligrams (mg), or micrograms (μg).

### 4.4. Saliva Sample Collection

Unstimulated saliva samples were collected in the morning after an overnight fast (12 h) at baseline using Oracollect-DNA kits (Genotek Inc., Ottawa, ON, Canada). Samples were centrifuged at 3000× *g* for 10 min at 4 °C following the manufacturer’s protocol. The supernatant was then transferred and stored at −80 °C until analysis.

### 4.5. DNA Extraction and Gene Copy Number Analysis Through qPCR

DNA was extracted from saliva samples using the Quick-DNA^TM^ Miniprep Plus Kit (Zymo Research, Freiburg, Germany) following the manufacturer’s instructions. DNA concentration and purity were assessed by spectrophotometry using a NanoDrop 2000c (Thermo Fisher Scientific, Waltham, MA, USA), based on absorbance values at 260 nm and A260/A280 and A260/A230 ratios. DNA samples were diluted to a final concentration of 10 ng/μL for use in quantitative polymerase chain reaction (qPCR). The *SFRS7* gene (arginine/serine-rich 7) was used as a reference gene due to its presence as a single copy in a haploid genome [23]. The 2 μL resulting DNA concentrated 10 ng/μL was used as template with 8 μL of SensiFAST SYBER Lo-ROX Kit (Nzytech, Lisboa, Portugal), and 500 nM of each specific primer for gene *AMY1* (Fw: 5′-GCGCTGGCAAAGGAGAGTTA, Rv: 5′-CCATCCAGTCCTCGTACTGC) or 250 nM in case of gene *SRSF7* (Fw: 5′-GTCCATGCTATTGTATTTACTTATACG, Rv: 5′-CCTTTCAGAGGTTTCAGCACATTA), with a total reaction volume of 16 μL. The qPCR reactions took place on Applied Biosystems 7500 Real-Time PCR System equipment (Applied Biosystems, Foster City, CA, USA) with the amplification program comprising 40 cycles, each consisting in an initial denaturation step at 95 °C for 15 s followed by 62 °C (for AMY1)/64 °C (for *SRSF7*) for 60 s for primers annealing and extension. These temperatures resulted from previous optimization steps. To assess the possibility of contamination and primer dimmers, no-template control samples (NTCs) were included for both pairs of primers. All samples were run in duplicate.

To determine the efficiency of the primers, a calibration curve was constructed for each gene (reference and target), considering six two-fold serial dilutions (1:2, 1:4, 1:8, 1:16, 1:32, 1:64) of a representative sample. Efficiency was calculated according to the expression below, where the slope of the standard curve was determined by the Applied Biosystems software v.1:

(1) $$E=\left(10^{\left(-\frac{1}{slope}\right)}-1\right)\;\times \;100$$

The specificity of the reactions was analyzed using a dissociation curve by adding an additional step at 95 °C during 15 s, followed by a constant increase in temperature between 60 °C and 95 °C. Data were obtained (quantitative cycle—Cq) using Applied Biosystems 7500Real-Time PCR software v1 (Applied Biosystems). Copy number values were obtained through relative quantification, by the 2^−ΔΔCT^ method [52,53].

### 4.6. Statistics

Statistical analyses were conducted using Statistical Package for the Social Sciences (SPSS) software (SPSS v.29 for Windows, IBM Software Group, Chicago, IL, USA). The normality of distributions was evaluated using the Shapiro–Wilk test (for *n* < 50). At baseline, participants were stratified into tertiles based on BMI z-score: Tertile 1 (<3.08), Tertile 2 (3.08–3.77), and Tertile 3 (>3.77). This stratification was applied to examine whether the associations between *AMY1* gene copy number, anthropometric variables, and dietary intake differed across increasing levels of adiposity. Since all children included in the study were classified as overweight or obese, the tertile division allowed us to capture gradients of excess weight within the sample. As well as, to assess the relationship between *AMY1* CNV and 9 months of intervention, participants were divided into two groups according to treatment.

Data are presented as means and standard deviations (SD). One-way ANOVA or Kruskal–Wallis tests were applied for group comparisons, followed by DMS post hoc tests when appropriate. Different superscript letters indicate statistically significant differences (*p* < 0.05). Pearson or Spearman correlation coefficients were calculated to assess the linear associations between salivary copies of *AMY1* and anthropometric or dietary variables. Partial correlations, controlling for gender, age, and dietary intake were also performed. Asterisks indicate significant correlations: * *p* < 0.05; ** *p* < 0.01.

## 5. Conclusions

The findings of this study highlight the interplay between childhood obesity, dietary intake, and salivary *AMY1* gene copy number. Higher *AMY1* copy numbers were positively associated with BMI z-score and carbohydrate intake, suggesting a potential genetic influence on dietary behavior and body composition. Although *AMY1* copy number was not directly associated with changes in BMI z-score during the intervention, it was linked to changes in PUFA intake, showing a possible role in modulating fat consumption.

The 9-month parent support program demonstrated to be effective in reducing BMI, BMI z-score, and energy and macronutrient intake among children with overweight and obesity. Although the number of copies of the gene *AMY1* was not observed to directly affect the efficacy of the intervention, in terms of anthropometric parameters, it appeared to have influence in some of the dietary changes induced by intervention, particularly reflected in fat intake. These findings underscore the importance of early, family-based lifestyle interventions for weight management and the potential influence of genetic factors such as *AMY1* CNV in shaping dietary responses and obesity risk.

Further research is warranted to explore the mechanisms through which *AMY1* expression affects metabolism and dietary behavior, and how these interactions may be leveraged to improve personalized intervention strategies for childhood obesity.

## Figures and Tables

**Table 1:** Characteristics and macronutrient intake of participants stratified by z-score BMI.

	Tertile 1 (<3.08) *n* = 30	Tertile 2 (3.08 to 3.77) *n* = 30	Tertile 3 (>3.77) *n* = 30	*p*-Value
	*n* (%)	*n* (%)	*n* (%)	
Boys	10 (31.0)	11 (36.7)	9 (30.0)	0.351
Girls	20 (69.0)	19 (63.3)	21 (70.0)	
	Mean (SD)	Mean (SD)	Mean (SD)	
Age (years)	5.3 (1.5)	5.7 (1.1)	5.1 (1.4)	0.185
Weight (kg)	27.0 (6.8) ^a^	34.8 (7.4) ^b^	37.6 (1.9) ^b^	<0.001
Height (cm)	116.1 (12.5)	120.8 (10.7)	116.6 (12.2)	0.240
Waist (cm)	66.5 (8.3) ^a^	74.0 (7.1) ^b^	79.0 (9.2) ^c^	<0.001
BMI (kg/m^2^)	19.8 (1.8) ^a^	23.5 (1.2) ^b^	27.1 (2.9) ^c^	<0.001
z-score BMI	2.2 (0.6) ^a^	3.4 (0.2) ^b^	4.3 (0.5) ^c^	<0.001
Energy (kcal)	1461.3 (368.4)	1678.2 (342.1)	1704.8 (434.0)	0.108
Carbohydrates (g)	167.7 (52.2)	193.4 (37.6)	196.3 (61.7)	0.197
Protein (g)	60.1 (14.7)	68.5 (14.4)	71.5 (26.7)	0.186
Fat (g)	54.7 (14.8)	60.6 (16.6)	61.4 (20.0)	0.423
SFA (g)	21.9 (7.0)	23.6 (7.5)	24.3 (6.2)	0.398
MUFA (g)	18.7 (3.1)	19.2 (3.9)	19.5 (4.1)	0.812
PUFA (g)	8.17 (3.6)	9.94 (3.8)	10.2 (4.0)	0.206

Abbreviations: BMI, body mass index; SFA, saturated fatty acids; MUFA, monounsaturated fatty acids; PUFA, polyunsaturated fatty acids; SD, standard deviation. Results are expressed as *n* (%) or mean (SD). Statistics realized by chi-square, one-way ANOVA or Kruskal–Wallis and post hoc DMS. Different letters mean significant difference when *p* < 0.05.

**Table 2:** Correlations between salivary copies of *AMY1*, anthropometry parameters and food intake.

	r	*p*
Anthropometry (*n* = 90)		
Weight	0.002	0.989
Waist	0.005	0.969
BMI	0.146	0.238
BMI z-score	0.253 *	0.039
Food intake (*n* = 90)		
Energy	0.226	0.116
Carbohydrates	0.125	0.374
Protein	0.146	0.298
Fat	0.258	0.063
SFA	0.232	0.094
MUFA	0.254	0.078
PUFA	0.291 *	0.034

Abbreviations: BMI, body mass index; SFA, saturated fatty acids; MUFA, monounsaturated fatty acids; PUFA, polyunsaturated fatty acids. Bivariate Correlation: (*) Indicates a correlation at *p* < 0.05. Regarding nutrient intake, *AMY1* copy number was positively and significantly correlated with PUFA intake (r = 0.291; *p* < 0.05). No significant correlations were observed for total energy intake or other macronutrients.

**Table 3:** Changes in anthropometric characteristics and food intake of children categorized by standard care and parent support program group after nine months of lifestyle intervention.

		Standard Care (Control) (*n* = 22)	Parent Support Program (*n* = 25)	*p*-Value
		Mean (SD)	Mean (SD)	
Weight (kg)	Baseline	31.5 (6.2)	33.4 (10.0)	0.462
	9 months	36.3 (7.9)	37.6 (11.9)	0.337
	Δ	4.8 (1.7)	4.2 (1.9)	0.070
Height (cm)	Baseline	118.4 (11.0)	118.4 (12.3)	0.477
	9 months	124.1 (10.5)	127.8 (10.9)	0.134
	Δ	5.7 (1.6)	9.4 (2.7)	0.115
Waist (cm)	Baseline	71.5 (7.2)	70.5 (10.0)	0.278
	9 months	72.6 (9.5)	69.8 (11.6)	0.215
	Δ	1.1 (2.3)	−0.07 (1.6)	0.307
BMI (kg/m^2^)	Baseline	22.5 (2.9)	23.3 (3.6)	0.586
	9 months	23.5 (3.9)	22.9 (4.1)	0.298
	Δ	1.06 (2.1)	−0.446 (1.4)	0.003 *
BMI z-score	Baseline	3.11 (1.0)	3.34 (1.0)	0.832
	9 months	3.34 (1.1)	3.14 (1.3)	0.277
	Δ	0.231 (0.4)	−0.203 (0.5)	<0.001 *
Energy (kcal)	Baseline	1620.9 (377.3)	1698.3 (481.5)	0.345
	9 months	1733.6 (393.1)	1443.8 (316.1)	0.022 *
	Δ	112.7 (46.6)	−254.5 (33.5)	0.032 *
Carbohydrates (g)	Baseline	185.2 (54.1)	204.5 (64.6)	0.326
	9 months	208.9 (61.0)	187.1 (50.3)	0.066
	Δ	23.7 (89.1)	−17.4 (68.6)	0.039 *
Protein (g)	Baseline	66.8 (20.1)	76.7 (33.0)	0.129
	9 months	70.5 (14.7)	63.8 (16.8)	0.264
	Δ	3.7 (33.9)	−12.9 (35.0)	0.079
Fat (g)	Baseline	66.0 (21.0)	61.7 (19.0)	0.372
	9 months	65.1 (21.9)	47.4 (13.4)	0.026 *
	Δ	−0.9 (5.5)	−14.3 (6.1)	0.045 *
SFA (g)	Baseline	24.3 (8.60	24.9 (8.1)	0.150
	9 months	23.2 (6.2)	18.6 (5.4)	0.130
	Δ	−1.1 (2.6)	−6.3 (3.6)	0.044 *
MUFA (g)	Baseline	20.1 (6.9)	20.9 (5.6)	0.237
	9 months	23.4 (9.2)	16.9 (6.1)	0.036 *
	Δ	3.3 (6.6)	−4.3 (5.9)	0.075
PUFA (g)	Baseline	10.8 (5.5)	8.95 (3.7)	0.036 *
	9 months	9.7 (4.4)	7.09 (2.7)	0.027 *
	Δ	−1.13 (7.0)	−1.86 (3.0)	0.260

* Significant differences for *p*-value < 0.05.

**Table 4:** Correlations between salivary copies of *AMY1* and anthropometry parameters and food intake changes in parent support program group after nine months of treatment.

	r	*p*
Anthropometry (*n* = 25)		
Δ Weight (kg)	0.038	0.885
Δ Waist (cm)	0.381	0.161
Δ BMI (kg/m^2^)	0.037	0.875
Δ BMI z-score	−0.008	0.972
Food intake (*n* = 25)		
Δ Energy (kcal)	−0.175	0.461
Δ Carbohydrates (g)	−0.124	0.604
Δ Protein (g)	−0.170	0.474
Δ Fat (g)	−0.421	0.064
Δ SFA (g)	−0.219	0.353
Δ MUFA (g)	−0.135	0.581
Δ PUFA (g)	−0.448	0.047

## Data Availability

There are restrictions on the availability of the data used for this trial due to the signed consent agreements around data sharing, which only allow access to external researchers for studies adhering to the project’s purposes. Requestors wishing to access the trial data used in this study can make a request to pep.tur@uib.es.

## Abbreviations

*AMY1*: *Amylase 1* gene; ANOVA: analysis of variance; BEDCA: Spanish Food Composition Database; BMI: Body mass index; CDC: Centers for Disease Control and Prevention; CNV: copy number variant; MUFA, monounsaturated fatty acids; NCT: no-template control samples; PUFA, polyunsaturated fatty acids; qPCR: quantitative polymerase chain reaction; SD: standard deviation; SFA, saturated fatty acids; SFRS7: arginine/serine-rich 7 gene; SNPs: single nucleotide polymorphisms; SPSS: Statistical Package for the Social Sciences; STOP: The Science and Technology in Childhood Obesity Policy project; WHO: World Health Organization.
